# Patient Perceptions of Chatbot Supervision in Health Care Settings

**DOI:** 10.1001/jamanetworkopen.2024.8833

**Published:** 2024-04-30

**Authors:** Jessica Ellis, Mika K. Hamer, Marlee Akerson, Matt Andazola, Annie Moore, Eric G. Campbell, Matthew DeCamp

**Affiliations:** 1 Center for Bioethics and Humanities, University of Colorado Anschutz Medical Campus, Aurora; 2 UCHealth, Denver, Colorado; 3 Division of General Internal Medicine, University of Colorado Anschutz Medical Campus, Aurora

## Abstract

This survey study assesses whether patients communicating with a chatbot in a large health care system were able to accurately identify it as an unsupervised computer application.

## Introduction

Conversational agents built on artificial intelligence (AI), known as chatbots, are being implemented for patient-facing communication in health care systems.^1^ Emerging research focuses on how users perceive chatbots’ anthropomorphic features^2^ and what qualities promote humanlike interactions.^3^ Limited evidence exists about patients’ understanding of chatbot supervision (ie, whether chatbots are operated or monitored by humans in real-time). To inform implementation of chatbots in health systems, we assessed patient perceptions of chatbot supervision in a health care setting.

## Methods

This survey study followed the American Association for Public Opinion Research (AAPOR) reporting guideline. We surveyed chatbot users who sent at least 3 messages back and forth with a large health system chatbot between July 2022 and September 2023. Internal electronic health record data suggested users would be predominantly White (76.8%); because of the chatbot avatar’s design, users may perceive the chatbot to be a White non-Hispanic female. Therefore, we designed a sampling strategy aimed at comparing perceptions of White non-Hispanic users to other races and ethnicities (not population-level estimates). Of the 65 de novo survey questions (eMethods in Supplement 1), 2 questions asked about perceived chatbot supervision. Participants were recruited by email, and they completed surveys and written consent online via REDCap. The study was approved by the Colorado Multiple Institutional Review Board.

Phase 1 (n = 142) used simple random sampling and revealed that most users and respondents were White. Therefore, to enable subgroup comparisons, in phases 2 (n = 298) and 3 (n = 177) we oversampled other races and ethnicities vs White non-Hispanic users in a 1:2 ratio.

We used multivariable logistic regression to estimate odds of correctly identifying the chatbot as an unsupervised software application, as a function of self-reported education, race and ethnicity, sex, income, and age. Two-sided *P* ≤ .05 was considered statistically significant. Analyses were conducted using Stata version 18.1 (StataCorp) from October to December 2023.

## Results

Among 617 surveys received from chatbot users (overall response rate: 20.0% [617 of 3089]), the mean (SD) age was 49.3 (16.8) years; 423 (68.6%) reported female sex and 166 (26.9%) reported male sex; 385 (62.4%) reported White non-Hispanic race and ethnicity and 231 (37.4%) reported other races and ethnicities; and 209 (33.9%) incorrectly classified the chatbot. When informed of lack of supervision, 24 chatbot users (11.5%) felt they had been tricked.

In the logistic regression model (Table), users with more than a 4-year degree had approximately 6 times higher odds (odds ratio [OR], 5.97 [95% CI, 3.03-11.74]; *P* < .001) of correctly identifying the chatbot as unsupervised compared with respondents with high school education or less. The odds of correctly identifying the chatbot as an unsupervised computer were approximately 1.6 times higher (OR, 1.60 [95% CI, 1.08-2.36]; *P* = .02) for White non-Hispanic users compared with other races and ethnicities (Figure).

**Table.** Multivariable Logistic Regression Results of Correctly Identifying Chatbot Supervision (N = 581)^a^

Respondent characteristics	OR (95% CI) [SE]	*P* value
Sex		
Male	1 [Reference]	NA
Female	0.74 (0.49-1.12) [0.16]	.16
Age, y		
18-34	1 [Reference]	NA
35-64	0.90 (0.55-1.47) [0.23]	.67
≥65	0.59 (0.32-1.10) [0.19]	.10
Income		
≤$13 590	1 [Reference]	NA
$13 591-$44 999	1.23 (0.71-2.14) [0.35]	.46
≥$45 000	1.41 (0.81-2.45) [0.40]	.22
Race and ethnicity		
Other race^b^ or Hispanic^c^	1 [Reference]	NA
White non-Hispanic	1.60 (1.08-2.36) [0.32]	.02
Education		
High school or less	1 [Reference]	NA
Some college	1.94 (1.14-3.31) [0.53]	.02
4-y college graduate	3.31 (1.81-6.07) [1.02]	<.001
More than 4-y degree	5.97 (3.03-11.74) [2.06]	<.001

Abbreviations: NA, not applicable; OR, odds ratio.

^a^ Thirty-six participants removed due to missingness.

^b^ Other race encompasses individuals who identified as the following race categories: American Indian or Alaska Native, Black or African American, Asian Indian, Chamorro, Chinese, Filipino, Japanese, Korean, Native Hawaiian, Other Asian, Other Pacific Islander, Samoan, Vietnamese.

^c^ Hispanic encompasses individuals who identified as the following ethnicity categories: Cuban; Mexican, Mexican American, Chicano; Other Hispanic, Latino, or Spanish origin; Puerto Rican.

**Figure.**
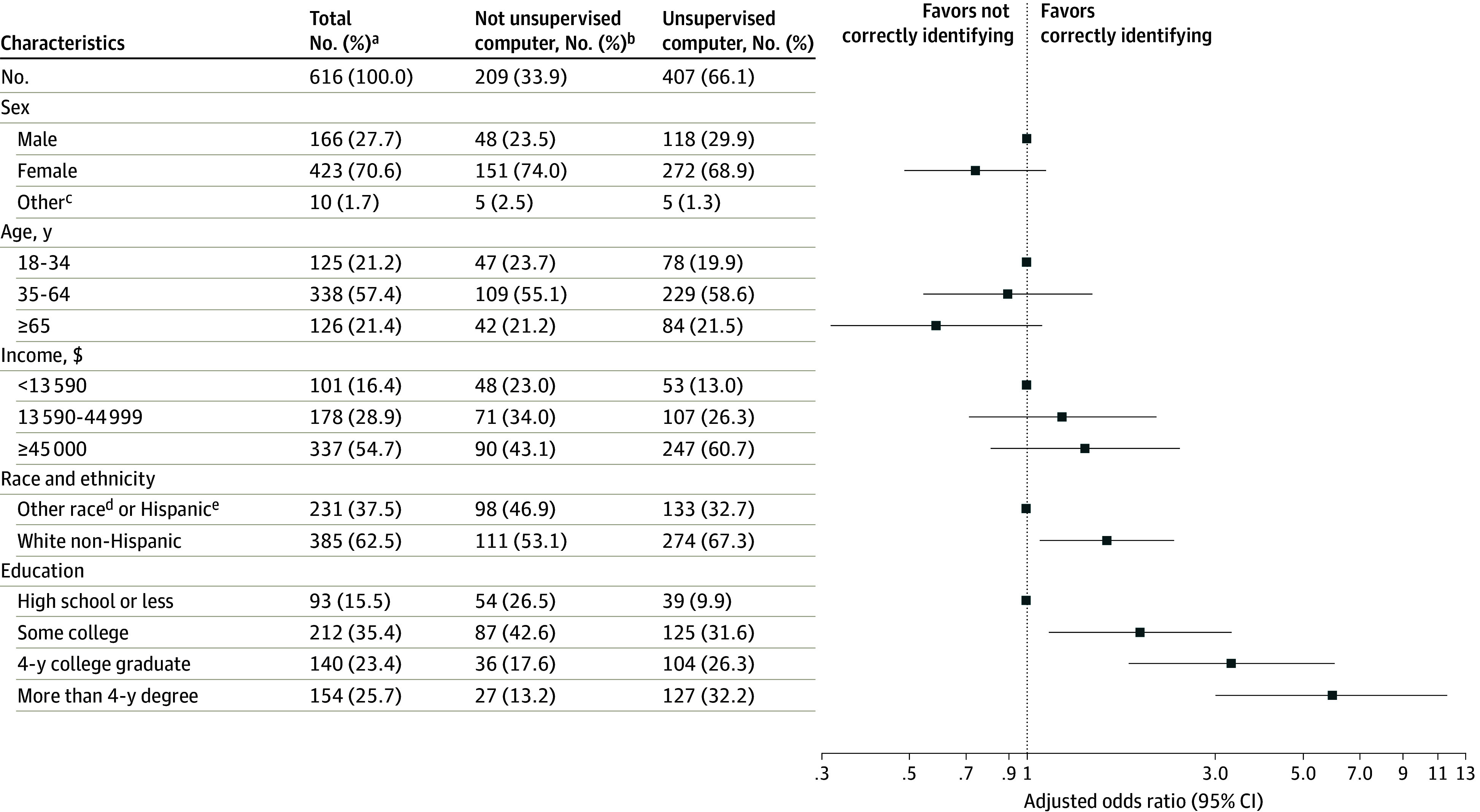
Forest Plot of Adjusted Odds Ratios of Correctly Identifying Chatbot Supervision Descriptive statistics and forest plot of adjusted odds ratios of correctly identifying chatbot supervision. ^a^“Not unsupervised computer” combines the following survey responses: 101 (48.3%) “computer with a person watching over in real time,” 34 (16.3%) “real person,” and 74 (35.4%) “I don't know.” ^b^Totals vary based on covariate missingness; 1 participant deleted due to missing outcome response. ^c^Other sex category was removed from regression analysis due to small sample size. ^d^Other race encompasses individuals who identified as the following race categories: American Indian or Alaska Native, Black or African American, Asian Indian, Chamorro, Chinese, Filipino, Japanese, Korean, Native Hawaiian, Other Asian, Other Pacific Islander, Samoan, Vietnamese. ^e^Hispanic encompasses individuals who identified as the following ethnicity categories: Cuban; Mexican, Mexican American, Chicano; Other Hispanic, Latino, or Spanish origin; Puerto Rican.

## Discussion

Most respondents identified the chatbot as an unsupervised computer. The chatbot disclosed being a “virtual assistant” during our study; despite this, some individuals were less likely to know it was unsupervised, with associations varying by education level and race and ethnicity. Fortunately, only a few felt tricked; nevertheless, adequate disclosure may be particularly important for groups marginalized by the health care system, for whom trust is already fragile, to avoid contributing to mistrust.

A dominant trend is to design chatbots with features that facilitate human-like interactions. Doing so may lead to mistaken identity for certain users, which could be particularly problematic as chatbots take on clinically related tasks.^4^ Recent federal policy emphasizes transparency and watermarking of AI-generated content.^5^ In other settings, people report similar levels of chatbot misidentification.^6^ Health care systems may need clearer messaging to ensure patients understand the purposes and functions of chatbots and whether they are supervised. Further research is needed to identify best practices for informing diverse patient users and explore how misperception of chatbots affects user trust, utilization, and satisfaction with chatbots and health care systems that use them.

Our study has limitations. Our sampling strategy may not generalize to the overall health care system’s patient population or other health care systems, or to the future in a rapidly changing AI environment.

## Notes

Supplement 1. **eMethods.** Chatbot Survey

Supplement 2.
Data Sharing Statement
