# Supplementary material for: Patient Perceptions of Chatbot Supervision in Health Care Settings
Source: JAMA Netw Open. 2024 Apr 30;7(4):e248833. doi: 10.1001/jamanetworkopen.2024.8833 (PMC11061768; doi:10.1001/jamanetworkopen.2024.8833)
Supplement: Supplement 1. — eMethods. Chatbot Survey [file jamanetwopen-e248833-s001.pdf]

Supplemental Online Content

Ellis J, Hamer MK, Akerson M, et al. Patient perceptions of chatbot supervision in health care settings. *JAMA Netw Open.* 2024;7(4):e248833.
doi:10.1001/jamanetworkopen.2024.8833

eMethods. Chatbot Survey

This supplemental material has been provided by the authors to give readers additional information about their work.

© 2024 Ellis J et al. *JAMA Network Open*

eMethods. Chatbot Survey

1. Our records indicate you recently interacted with Livi on the UCHealth website. Do you remember this interaction?
    a. Yes
    b. No
2. How often do you interact with chatbots such as Amazon's Alexa, Apple's Siri, or other chatbots?
    a. Often (more than once a week)
    b. Sometimes (once a month)
    c. Rarely (a few times per years)
    d. Never
3. In general, how would you rate your trust or distrust of chatbots like Alexa or Siri?
    a. I completely trust chatbots
    b. I trust chatbots a little
    c. I distrust chatbots a little
    d. I completely distrust chatbots
    e. I don't know
4. In your most recent communication with Livi, what were you trying to do? (Check each that applies to you)
    a. Get an appointment with my doctor/one of my doctors
    b. Get an appointment with a doctor you had not seen before
    c. Check on an existing appointment
    d. Get a prescription refilled
    e. Get the results of a medical test
    f. Get advice about a health issue
    g. Get information about a bill
    h. Other [BRANCH TO: question 5]
5. [BRANCHED QUESTION] Please explain if you selected "Other"
    a. Overall, how would you rate your interaction with Livi?
    b. Very helpful
    c. Mostly helpful
    d. Mostly unhelpful
    e. Very helpful
6. How would you rate your understanding of what Livi wrote?
    a. I understood Livi completely
    b. I understood most of what Livi said
    c. I understood only a little of what Livi said
    d. I did not understand Livi at all
    e. I don't know/unsure
7. How would you rate Livi's understanding of what you wrote?
    a. Livi understood me completely
    b. Livi understood most of what I said
    c. Livi understood only a little of what I said
    d. Livi did not understand me at all
    e. I don't know/unsure
8. Which of the following are reasons you decided to interact with Livi? Check all that apply:
    a. I wanted an answer right away
    b. I wasn't sure I could get my doctor or nurse on the phone
    c. I had a simple question that I thought Livi could answer
    d. I felt like I had to communicate with Livi
    e. I don't like talking to people on the phone
    f. I was worried I would be charged if I called my doctor's office
    g. I was curious to see what Livi could do
9. When you were communicating with Livi did you think Livi was...
    a. A real person [BRANCH TO: question 10]
    b. A computer with a person watching over Livi in real time [BRANCH TO: question 11]

    c. A computer acting on its own, without anyone watching over Livi
    d. I don't know [BRANCH TO: question 10]
10. [BRANCHED QUESTION] Livi is actually a computer program. No one helps Livi in real time. Knowing this now, do you feel tricked?
    a. Yes
    b. No
11. [BRANCHED QUESTION] Livi is a computer program, but no one helps Livi in real time. Knowing this now, do you feel tricked?
    a. Yes
    b. No
12. To what extent do you think Livi is intended to?
    a. Help patients like you navigate the uchealth system?
    b. Help patients like you access information quickly?
    c. Limit your access to doctors and nurses?
    d. Save money by not having to pay people to interact with users in real-time?
    e. Make life easier for healthcare workers?
    f. Collect information about you?
    g. Make life easier for you?
13. To what extent do you think each of the following benefit from Livi the Chatbot?
    a. Your care team
    b. The UCHealth System
    c. Your health insurance company
    d. A health technology company
14. Rate your disagreement/agreement with the following statements:
    a. My doctor gets the information I tell Livi.
    b. My care team reviews the information Livi gives me to ensure it is right.
    c. There is a record of what information Livi gives me in case something happens.
    d. The information Livi gives is specific to me and my health needs
    e. I would tell a chatbot like Livi and my doctor different things
    f. Livi will tell me the same information as my doctor
15. How much do you agree or disagree with the following statement: It seemed like Livi wanted me to do something I did not really want to do.
    a. Strongly Disagree
    b. Disagree
    c. Neutral
    d. Agree
    e. Strongly Agree
16. Now we are going to ask some questions about Livi's appearance and how that might have impacted your interaction with Livi, or any future interactions you may have with Livi. Do you think Livi is...
    a. White
    b. Black or African American
    c. American Indian or Alaska Native
    d. Asian
    e. Native Hawaiian or Other Pacific Islander
    f. Other [write in]
    g. None of these
    h. I don't know/can't tell
17. What gender do you think Livi is?
    a. Male
    b. Female
    c. Transgender male
    d. Transgender female
    e. Gender non-conforming
    f. I don't know/can't tell
18. If you could control what Livi looked like, what would you do?
    a. I want Livi to look like a cartoon not like a real person

b. I want Livi to look like a real person
c. I don't care what Livi looks like

19. If you could control what Livi looked like, what would you do? (Choose all that apply)
    a. I want Livi to look like me in terms of gender
    b. I want Livi to look like me in terms of race
    c. I want Livi to look like my doctor
    d. I don't care what Livi looks like
20. Livi can see your medical record. How worried are you about this?
    a. I am very worried about this.
    b. I am somewhat worried about this.
    c. I am a little worried about this.
    d. I am not at all worried about this.
21. Overall, what do you think about the privacy of the information you share with Livi?
    a. I am very worried about privacy
    b. I am somewhat worried about privacy
    c. I am a little worried about privacy
    d. I am not at all worried about privacy
22. How much do you disagree/agree with this statement: The information I give Livi could be used against me.
    a. Strongly Disagree
    b. Disagree
    c. Neutral
    d. Agree
    e. Strongly Agree
23. How concerned are you about the privacy of personal information you give Livi, compared to other information you give on the internet?
    a. I am less concerned about privacy with Livi than the Internet
    b. I am somewhat less concerned about privacy with Livi than the Internet
    c. I have similar concern about privacy with Livi than the Internet
    d. I am somewhat more concerned about privacy with Livi than the Internet [BRANCH TO: question 24]
    e. I am much more concerned about privacy with Livi than the Internet [BRANCH TO: question 24]
24. [BRANCHED QUESTION] If you are more concerned about the privacy of personal information you give Livi, compared to the Internet, what is reason?
    a. It's not safe, someone could steal my information
    b. I don't know how the information would be used
    c. I don't know who I'm dealing with
    d. I'm unfamiliar with how the technology works
    e. Other
25. Could you ever imagine a time you might give Livi incorrect information, for any reason whatsoever?
    a. Yes [BRANCH TO: question 26]
    b. No
26. [BRANCHED QUESTION] If yes, For what reason might you give Livi incorrect information?
    a. To protect my own identity.
    b. Just out of curiosity, to see what Livi would do.
    c. To save time.
    d. Out of concern my information could be stolen.
    e. Other
27. Trust is very important in healthcare. We want to understand your relationship with trust in medicine generally and in regard to Livi specifically. Rate your disagreement/agreement with the following statements:
    a. You'd better be cautious when dealing with health care organizations
    b. Patients have sometimes been deceived or misled by health care organizations
    c. When health care organizations make mistakes, they usually cover it up
    d. Health care organizations have sometimes done harmful experiments on patients without their knowledge

  e. Health care organizations don't always keep your information totally private
  f. Sometimes I wonder if health care organizations really know what they are doing
  g. Mistakes are common in health care organizations
  h. Sometimes my doctor cares more about what is convenient for (him/her) than about my medical needs.
  i. My doctor is extremely thorough and careful.
  j. I completely trust my doctor's decisions about which medical treatments are best for me.
  k. My doctor is totally honest in telling me about all of the different treatment options available for my condition.
  l. All in all, I have complete trust in my doctor.

28. If you could choose, would you prefer to be treated by a doctor who is...?
  a. Male
  b. Female
  c. It doesn't matter
  d. Don't know
29. If you could choose, would you prefer to be treated by a doctor who is...?
  a. Your own race or ethnic group
  b. Another race or ethnic group
  c. It doesn't matter
  d. Don't know
30. Overall, how much do you trust Livi?
  a. I completely trust Livi
  b. I mostly trust Livi
  c. I trust Livi a little
  d. I don't trust Livi at all
31. To what extent do you think the information Livi gives you is accurate?
  a. To a great extent
  b. To some extent
  c. Very little
  d. Not at all
32. How much do you disagree/agree with this statement: Livi will make recommendations about my health care that really should be made by a real person, such as a doctor(s) or a nurse.
  a. Strongly Disagree
  b. Disagree
  c. Neutral
  d. Agree
  e. Strongly Agree
33. Knowing that Livi the chatbot is being used, do you
  a. Trust your doctor(s) a lot less
  b. Trust your doctor(s) a little less
  c. Trust your doctor(s) a little more
  d. Trust your doctor(s) a lot more
  e. Knowing this does not change how much I trust my doctor.
34. Do you...
  a. Trust Livi more than your doctor(s)
  b. Trust Livi and your doctor(s) the same
  c. Trust Livi less than your doctor(s)
35. When you interact with your doctor, how worried are you that your doctor might judge or think badly of you, based on what you said?
  a. Very worried
  b. Somewhat worried
  c. A little worried
  d. Not at all worried
36. When you interact with Livi, how worried are you that your doctor might judge or think badly of you, based on what you said to Livi?
  a. Very worried

© 2024 Ellis J et al. *JAMA Network Open*

   b. Somewhat worried
   c. A little worried
   d. Not at all worried
37. To what extent do you feel Livi limits your access to health care professionals?
    a. To a great extent
    b. To some extent
    c. Very little
    d. Not at all
38. Do you think you would have had a better experience with Livi if you were a different race?
    a. Yes
    b. No
    c. I'm not sure
39. Do you think you would have had a better experience with Livi if you were a different gender?
    a. Yes
    b. No
    c. I'm not sure
40. Do you think Livi interacts with all patients in the same way regardless of their race and ethnicity?
    a. Yes
    b. No
    c. I'm not sure
41. Do you think Livi interacts with all patients in the same way regardless of their gender?
    a. Yes
    b. No
    c. I'm not sure
42. Do you think Livi interacts with all patients in the same way regardless of what language they speak?
    a. Yes
    b. No
    c. I'm not sure
43. Have you ever interacted with Livi related to advanced care planning or power of attorney forms?
    a. Yes
    b. No
44. What is the zip code of your current residence?
45. Date of Birth
46. What is your sex?
    a. Male
    b. Female
    c. Other
47. Are you Hispanic, Latino, or of Spanish Origin?
    a. No: not Hispanic, Latino, or Spanish Origin
    b. Yes: Mexican, Mexican Am., Chicano
    c. Yes: Puerto Rican
    d. Yes: Cuban
    e. Yes: Another Hispanic, Latino, or Spanish origin
48. What is your race?
    a. White
    b. Black or African American
    c. American Indian or Alaska Native
    d. Chinese
    e. Filipino
    f. Asian Indian
    g. Vietnamese
    h. Korean
    i. Japanese
    j. Other Asian
    k. Native Hawaiian
    l. Samoan

   m. Chamorro
   n. Other Pacific Islander
49. Are you currently covered for any of the following types of health insurance or health coverage plans?
    a. Insurance through a current or former employer or union
    b. Insurance purchased directly from an insurance company
    c. Medicare
    d. Medicaid, Medical Assistance, or any other kind of government-assistance plan for those with low incomes or disability
    e. TRICARE
    f. VA
    g. Indian Health Service
    h. Other
50. What was your total income during the past 12 months?
    a. Less than $13,590
    b. $13,591 to $44,999
    c. $45,000 to $139,999
    d. $140,000 to $149,999
    e. $150,000 to $199,999
    f. $200,000+
51. What is the highest degree or level of school you have completed?
    a. 8th grade or less
    b. Some high school, but did not graduate
    c. High school graduate or GED
    d. Some college or 2-year degree
    e. 4-year college graduate
    f. More than 4-year college degree
52. Using any number from 0 to 10, where 0 is the worst health system possible and 10 is the best health system possible, what number would you use to rate your health system?
53. In general, how would you rate your overall health?
    a. Excellent
    b. Very good
    c. Good
    d. Fair
    e. Poor
54. How well do you speak English?
    a. Very well
    b. Well
    c. Not well
55. Do you have difficulty hearing, even if using a hearing aid(s)?
    a. No difficulty
    b. Some difficulty
    c. A lot of difficulty
    d. Cannot do at all
56. Do you have difficulty seeing, even if wearing glasses?
    a. No difficulty
    b. Some difficulty
    c. A lot of difficulty
    d. Cannot do at all
57. Do you have difficulty remembering or concentrating?
    a. No difficulty
    b. Some difficulty
    c. A lot of difficulty
    d. Cannot do at all
58. Do you have difficulty walking or climbing steps?
    a. No difficulty
    b. Some difficulty

© 2024 Ellis J et al. *JAMA Network Open*

c. A lot of difficulty
d. Cannot do at all

59. Do you have difficulty with self-care, such as washing all over or dressing?
    a. No difficulty
    b. Some difficulty
    c. A lot of difficulty
    d. Cannot do at all
60. In your usual language, do you have difficulty communicating, for example understanding or being understood?
    a. No difficulty
    b. Some difficulty
    c. A lot of difficulty
    d. Cannot do at all
61. How often do you need to have someone help you when you read instructions, pamphlets, or other written material from your doctor or pharmacy?
    e. Never
    f. Rarely
    g. Sometimes
    h. Often
    i. Always
62. Overall, how often do you use the internet?
    j. Never
    k. Less than once a week
    l. Once a week
    m. Several times a week
    n. At least once a day
    o. Multiple times a day
    p. Most of the day
63. Overall, how confident do you feel using computers, smartphones, or other electronic to do the things you need to do online?
    q. Not at all confident
    r. Only a little confident
    s. Somewhat confident
    t. Very confident
64. Would you like to receive the $20 compensation for participating today? If yes, please enter your email address below.
65. Would you be interested in participating in an in-depth interview to talk more about Livi and your answers? You will receive $100 for your time.
    u. Yes
    v. No

© 2024 Ellis J et al. *JAMA Network Open*
