# Supplementary material for: Patient Perceptions of Chatbot Supervision in Health Care Settings
Source: JAMA Netw Open. 2024 Apr 30;7(4):e248833. doi: 10.1001/jamanetworkopen.2024.8833 (PMC11061768; doi:10.1001/jamanetworkopen.2024.8833)
Supplement: Supplement 2. — Data Sharing Statement [file jamanetwopen-e248833-s002.pdf]

# Data Sharing Statement

Ellis. Patient Perceptions of Chatbot Supervision in Health Care Settings. *JAMA Netw Open*. Published April 30, 2024. doi:10.1001/jamanetworkopen.2024.8833

**Data**
**Data available:** Yes
**Data types:** Deidentified participant data
**How to access data:** Email matthew.decamp@cuanschutz.edu
**When available:** beginning date: 06-30-2024

**Supporting Documents**
**Document types:** None

**Additional Information**
**Who can access the data:** Researchers whose proposed use of the data has been approved
**Types of analyses:** For any purpose
**Mechanisms of data availability:** After approval of a proposal
